# Repair of sub-lethal freezing damage in leaves of *Arabidopsis thaliana*

**DOI:** 10.1186/s12870-020-2247-3

**Published:** 2020-01-20

**Authors:** Kora Vyse, Johanna Penzlin, Kjell Sergeant, Dirk K. Hincha, Rajeev Arora, Ellen Zuther

**Affiliations:** 1 Max-Planck-Institut für Molekulare Pflanzenphysiologie, Am Mühlenberg 1, 14476 Potsdam, Germany; 2 Environmental Research and Innovation (ERIN) Department, Luxembourg Institute of Science and Technology (LIST), 5 Avenue des Hauts-Fourneaux, L-4362 Esch/Alzette, Luxembourg; 3 Department of Horticulture, Iowa State University, Ames, Iowa 50010 USA

**Keywords:** Freezing damage, Repair, Arabidopsis, Respiration, Photosynthesis, Gene expression

## Abstract

**Background:**

The detrimental effects of global climate change direct more attention to the survival and productivity of plants during periods of highly fluctuating temperatures. In particular in temperate climates in spring, temperatures can vary between above-zero and freezing temperatures, even during a single day. Freeze-thaw cycles cause cell membrane lesions that can lead to tissue damage and plant death. Whereas the processes of cold acclimation and freeze-thaw injury are well documented, not much is known about the recovery of plants after a freezing event. We therefore addressed the following questions: i. how does the severity of freezing damage influence repair; ii. how are respiration and content of selected metabolites influenced during the repair process; and iii. how do transcript levels of selected genes respond during repair?

**Results:**

We have investigated the recovery from freezing to sub-lethal temperatures in leaves of non-acclimated and cold acclimated *Arabidopsis thaliana* plants over a period of 6 days. Fast membrane repair and recovery of photosynthesis were observed 1 day after recovery (1D-REC) and continued until 6D-REC. A substantial increase in respiration accompanied the repair process. In parallel, concentrations of sugars and proline, acting as compatible solutes during freezing, remained unchanged or declined, implicating these compounds as carbon and nitrogen sources during recovery. Similarly, cold-responsive genes were mainly down regulated during recovery of cold acclimated leaves. In contrast, genes involved in cell wall remodeling and ROS scavenging were induced during recovery. Interestingly, also the expression of genes encoding regulatory proteins, such as 14–3-3 proteins, was increased suggesting their role as regulators of repair processes.

**Conclusions:**

Recovery from sub-lethal freezing comprised membrane repair, restored photosynthesis and increased respiration rates. The process was accompanied by transcriptional changes including genes encoding regulatory proteins redirecting the previous cold response to repair processes, e.g. to cell wall remodeling, maintenance of the cellular proteome and to ROS scavenging. Understanding of processes involved in repair of freeze-thaw injury increases our knowledge on plant survival in changing climates with highly fluctuating temperatures.

## Background

Effects of global climate change, such as milder winters and higher temperature fluctuations in spring in the Northern parts of the world strongly impact the survival of plants due to late-season cold spells. Cold acclimation, which increases freezing tolerance of plants during exposure to low, but non-freezing temperatures, improves winter survival [1]. Most plants native to temperate and boreal climates undergo an annual cycle of acclimation and deacclimation to increase freezing tolerance in fall and reduce tolerance in spring [2, 3].

Highly fluctuating temperatures result in frequent freeze-thaw cycles, causing membrane lesions by membrane contraction and expansion [4, 5], affecting plant productivity and resulting in economic losses for agriculture [6]. Extensive frost damage in spring after a warm spell has been documented repeatedly [7–10].

Since tissues are injured by both freeze-induced dehydration and rehydration during thawing [1, 11], the term “freeze-thaw stress tolerance” (FTST) was defined to describe the ability of a plant to survive a freeze-thaw cycle and to recover from non-lethal lesions after thawing [12, 13]. Whereas freeze-thaw injury has been investigated in detail, little attention was paid to the ability of injured tissue to recover from freeze-thaw stress, although this is critical for FTST [14–16]. Freeze-thaw injury is mainly caused by extracellular ice formation provoking freeze-dehydration which results in membrane destabilization [5]. Though the plasma membrane is considered as the primary site of injury [17], there is also extensive damage to the chloroplast envelope and thylakoid membranes [18] leading to decreased photosynthetic activity [19]. In addition, some chloroplast stromal enzymes such as Rubisco are inactivated during freezing [20].

Physiological and molecular processes involved in post-thaw recovery from non-lethal lesions, critical for frost survival, have only scarcely been investigated. During post-thaw recovery of injured tissues, membrane damage is reversed and turgor regained, e.g. during full recovery of onion cells [21]. Proteomic studies in onion showed that proteins responsible for cell repair, affecting reactive oxygen species (ROS) scavenging, removal and assembly of denatured proteins, membrane and cell wall stabilization and restoration of the cellular ionic environment accumulated during post-thaw recovery [13]. Another study in spinach supported these findings, with post-thaw recovery accompanied by a reduction in ion leakage, recovery of photosystem II efficiency, activation of antioxidant enzymes and dissipation of ROS [22].

Compatible solutes such as sugars or proline that are known to stabilize membranes during freezing accumulate during cold acclimation and decrease during deacclimation [23, 24]. They might also play a role for the level of freeze-thaw damage and post-thaw recovery. They furthermore increase the osmotic potential of the cells, stabilize proteins during freezing [2, 25] and act as ROS scavengers [26]. Metabolic changes in *Avena sativa* crowns recovering from freezing over 14 days included increased amounts of amino acids and decreased amounts of sucrose, fructose and TCA cycle intermediates [27].

In the present paper, we have quantified repair processes after a freeze-thaw cycle at the plasma membrane using an electrolyte leakage assay and at the chloroplast using chlorophyll fluorescence measurements in leaves of non-acclimated and cold acclimated Arabidopsis plants. In addition, we determined respiratory activity and investigated pool sizes of proline, glucose, fructose, sucrose and raffinose as indicators of metabolic activity during recovery. The expression of genes orthologous to genes encoding proteins previously identified as significantly altered in abundance during recovery in onion [13] was investigated by qRT-PCR. The results provide new insights into the repair process after sub-lethal freeze-thaw injury in Arabidopsis and identify transcriptional regulation to be important for post-thaw recovery.

## Results

### Sub-lethal freezing injury is quickly repaired after thawing

**Fig. 1:**
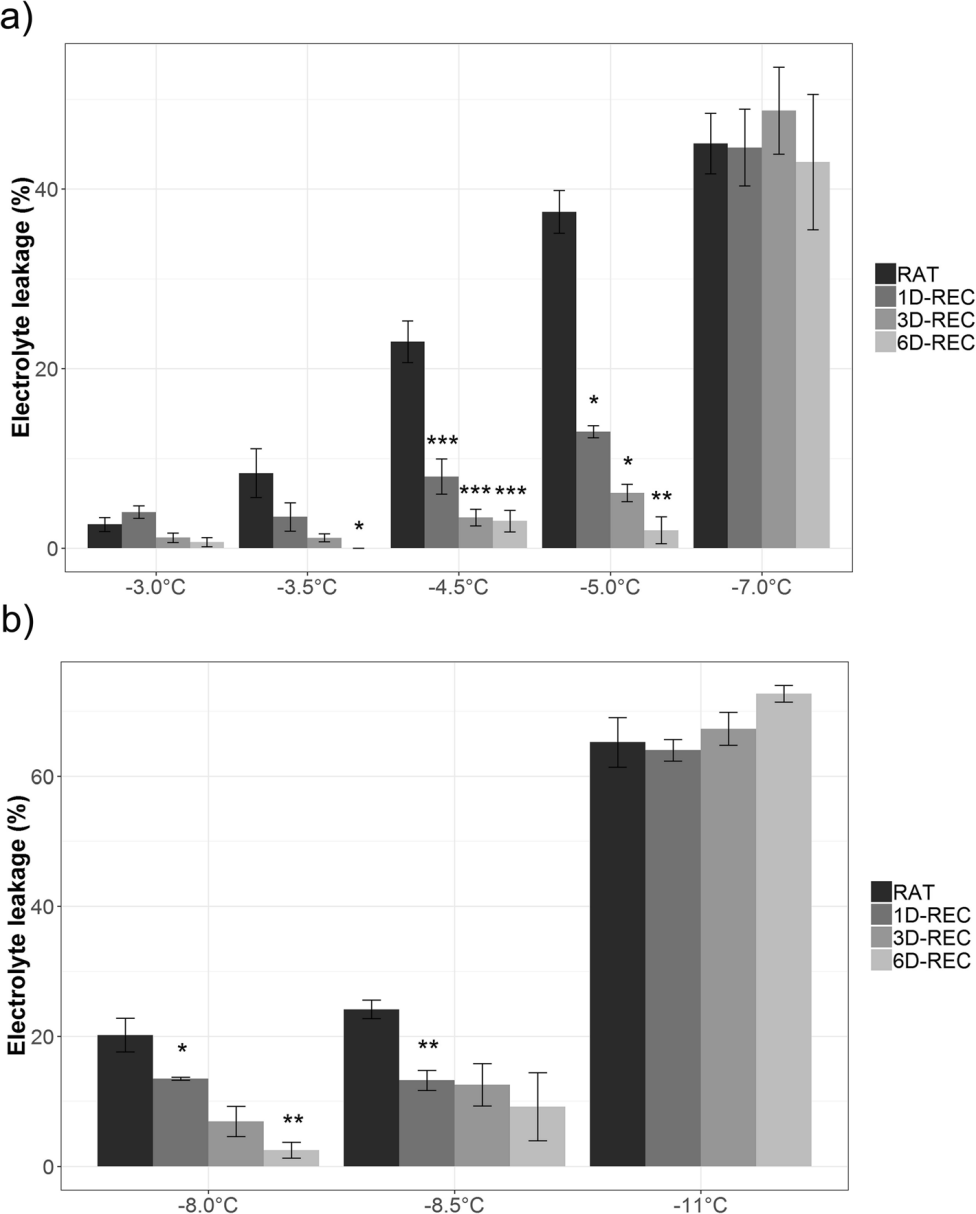
Relative electrolyte leakage values of Arabidopsis leaves frozen to different temperatures right after thawing (RAT) or after recovery for 1 day (1D-REC), 3 days (3D-REC) or 6 days (6D-REC) at 4 °C for **a** non-acclimated plants and **b** cold acclimated plants. Averages of four replicates are shown with standard errors. Significance levels between values measured at RAT and at the subsequent time points determined by Student’s t-test are indicated as: ***, *p* < 0.001, **, *p* < 0.01, *, *p* < 0.05

Freeze-thaw injury and post-thaw recovery were analyzed for fully developed leaves by electrolyte leakage measurements after freezing to five different temperatures (Table 1) for non-acclimated (NA) and three temperatures for cold-acclimated (ACC) *Arabidopsis thaliana* Col-0 plants. Freezing temperatures slightly higher (warmer) than the previously reported LT_50_ values of − 5.34 °C NA and − 9.68 °C ACC [28] were chosen to generate non-lethal lesions in contrast to irreversible injury after freezing to temperatures below (colder than) the respective LT_50_. Electrolyte leakage was measured in samples right after thawing at 4 °C (RAT) or at 1D-REC, 3D-REC and 6D-REC at 4 °C. Recovery at 20 °C caused unreliable electrolyte leakage values due to bacterial growth (not shown) and was therefore not further investigated. At mild freezing temperatures, such as − 3 °C, electrolyte leakage at RAT was very low (2.65%), but increased gradually with decreasing temperatures to 45.10% when non-acclimated leaves were frozen to − 7 °C (Fig. 1a).

**Table 1:** Treatment temperatures of leaves for different measurements. Freezing temperatures were chosen according to previously reported LT_50_ values of − 5.34 °C NA and − 9.68 °C ACC [28]

Method	Non-acclimated		Cold acclimated	
	Samples frozen to		Samples frozen to	
	non-lethal temperature (°C)	lethal temperature (°C)	non-lethal temperature (°C)	lethal temperature (°C)
Electrolyte leakage	− 3.0, − 3.5, − 4.5, − 5	− 7	− 8, − 8.5	− 11
Chlorophyll fluorescence imaging	− 5, − 6, − 7	− 8	− 9, − 10, − 11	− 12, − 13
Respiration	− 6, − 7	− 8	− 8, − 9	− 12
Sugar content	− 4.5, − 5, − 6, − 7	− 8	− 8, − 8.5, − 9, − 10, − 11	− 12, − 13
Proline content	− 4.5, − 5, − 6, − 7	− 8	− 8, − 8.5, − 9, − 10, − 11	− 12, − 13
Gene expression analysis (qRT-PCR)	− 5, − 6, − 7	− 8	− 9, − 10, − 11,	− 12, − 13

Already after 1D-REC electrolyte leakage was significantly decreased and it decreased further over 6D-REC even in leaves frozen to − 5 °C. No recovery from freeze-thaw injury was observed after freezing to − 7 °C. A similar pattern was observed for leaves of cold acclimated plants (Fig. 1b) with increasing injury at RAT conditions following exposure to decreasing freezing temperatures and a recovery process over 6D-REC when the freezing temperature was above the previously reported LT_50_ ACC. At a freezing temperature below the LT_50_ ACC (− 11 °C), no recovery was observed and electrolyte leakage values actually increased from 65.2% at RAT to 72.7% 6D-REC.

**Fig. 2:**
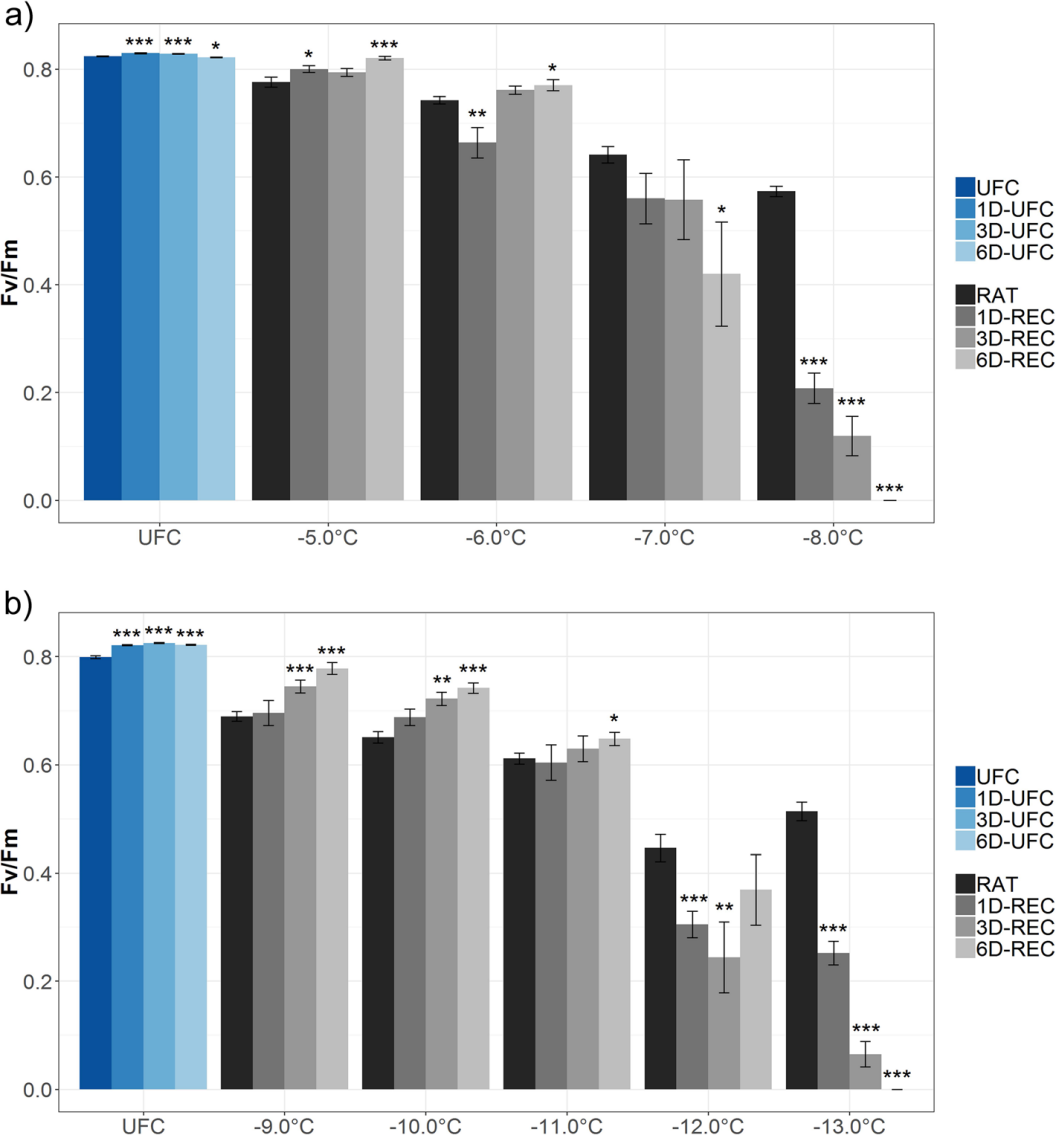
Maximum quantum yield efficiency of PSII (Fv/Fm) in unfrozen Arabidopsis leaves (UFC, 1D-UFC, 3D-UFC, 6D-UFC) and in leaves frozen to different temperatures right after thawing (RAT) or after recovery for 1 day (1D-REC), 3 days (3D-REC) or 6 days (6D-REC) at 4 °C for **a** non-acclimated plants and **b** cold acclimated plants. Control samples were incubated at 4 °C for the respective times. Three independent experiments were carried out for each temperature except for − 5 °C, − 7 °C, − 11 °C and − 12 °C with only one and − 13 °C with two experiments. Data are means of 10 to 15 biological replicates per experiment with standard errors. Significance levels between values measured at UFC/RAT and at the subsequent time points determined by Student’s t-test are indicated as: ***, *p* < 0.001, **, *p* < 0.01, *, *p* < 0.05

Chlorophyll fluorescence imaging measurements were performed with leaves from non-acclimated and cold-acclimated plants to reveal potential repair of freezing damage in chloroplasts. For these experiments, lower freezing temperatures than for the electrolyte leakage measurements were used (− 5 °C to − 8 °C for non-acclimated and − 9 °C to − 13 °C for acclimated leaves). Chlorophyll fluorescence imaging consistently yields lower (more negative) LT_50_ values than electrolyte leakage assays [19] arguably because the preincubation in water before the conductivity measurements causes secondary damage to the leaves, resulting in greater ion leakage and thus a slight underestimation of LT_50_. Decreasing Fv/Fm ratios represent a decreasing maximum quantum yield efficiency of PSII and therefore reflect damage to the thylakoid membranes. When non-acclimated leaves were frozen to decreasing temperatures, the Fv/Fm ratio at RAT decreased from 0.78 (at − 5 °C) to 0.57 (at − 8 °C) compared to 0.82 in the unfrozen control (UFC) leaves (Fig. 2a). Fv/Fm ratio at 6D-REC increased to values similar to control conditions when the leaves were frozen to − 5 °C. Also, leaves frozen to − 6 °C were able to recover after an initial additional drop of the Fv/Fm ratio at 1D-REC resulting in values above the RAT at 6D-REC but slightly below the respective control. At lower freezing temperatures, the initial injury was not reversed and the Fv/Fm ratio decreased further to 0.42 (− 7 °C) or even to 0 (− 8 °C).

Leaves from cold acclimated plants showed a similar decline in the Fv/Fm ratios at RAT conditions with decreasing freezing temperatures (Fig. 2b). UFC leaves showed a maximum quantum yield of 0.80 to 0.82. Leaves frozen to − 9 °C or − 10 °C displayed a continuous increase in Fv/Fm during 6D-REC from 0.69 to 0.78 or from 0.65 to 0.74, respectively. Leaf samples frozen to − 11 °C still showed a small increase in the Fv/Fm ratio at 6D-REC. Samples exposed to lower freezing temperatures showed a decrease of the Fv/Fm values over time which reached zero at 6D-REC after freezing to − 13 °C.

### Respiration rate is increased during recovery

**Fig. 3:**
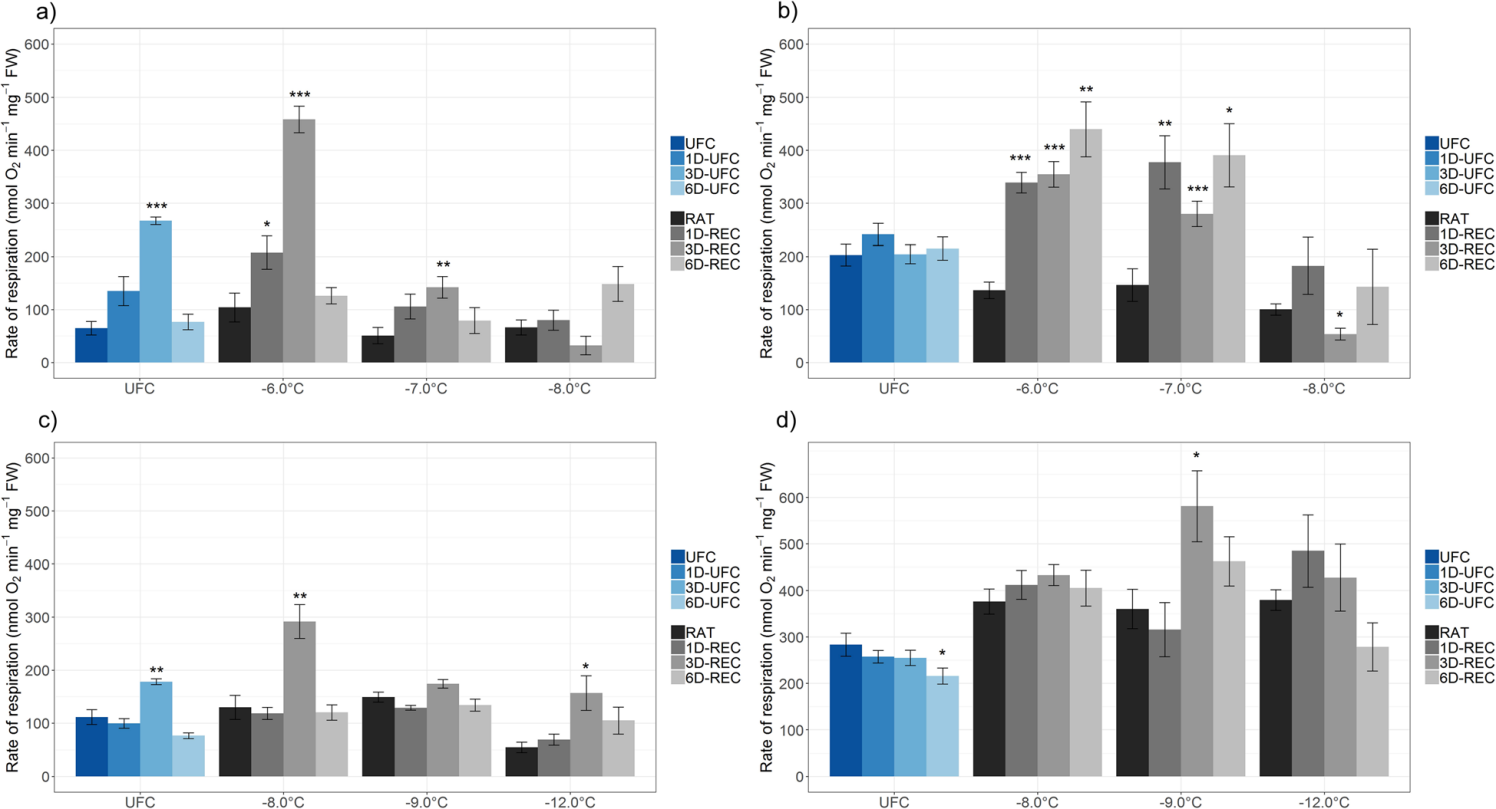
Respiration rates measured as oxygen consumption in unfrozen Arabidopsis leaves (UFC, 1D-UFC, 3D-UFC, 6D-UFC) and in leaves frozen to different temperatures right after thawing (RAT) or after recovery for 1 day (1D-REC), 3 days (3D-REC) or 6 days (6D-REC) at 4 °C for **a**, **b** non-acclimated plants and **c**, **d** cold acclimated plants. **a**, **c** Respiration was measured at the recovery temperature (4 °C); **b**, **d** respiration was measured at room temperature (21 °C). Data are the means of five replicates with two leaf discs each with standard errors. Significance levels between values measured at UFC/RAT and at the subsequent time points determined by Student’s t-test are indicated as: ***, *p* < 0.001, **, *p* < 0.01, *, *p* < 0.05

Oxygen consumption is highly influenced by the temperature at which respiration rates are measured [29]. Since the recovery process took place at 4 °C, we measured respiration rates during recovery at that temperature (Fig. 3a, c). In addition, measurements were also performed at 21 °C (Fig. 3b, d), a temperature more generally used for such measurements. Samples from non-acclimated and cold acclimated plants were frozen to either − 6 °C, − 7 °C and − 8 °C, or to − 8 °C, − 9 °C and − 12 °C, respectively. Higher respiration rates were measured in all samples processed at 21 °C compared to 4 °C, with an average over all samples of non-acclimated plants of 238 nmol O_2_ min^− 1^ mg^− 1^ FW versus 134 nmol O_2_ min^− 1^ mg^− 1^ FW at 4 °C and over all respective samples of cold acclimated plants of 370 nmol O_2_ min^− 1^ mg^− 1^ FW versus 131 nmol O_2_ min^− 1^ mg^− 1^ FW at 4 °C.

Measurements at 4 °C revealed increased respiration rates during the recovery time in UFC samples, in particular for non-acclimated leaves (Fig. 3a). In addition, when non-acclimated samples were frozen to − 6 °C, or acclimated samples to − 8 °C, respiration rates increased 4.4-fold (Fig. 3a) or 2.2-fold (Fig. 3c) during recovery at 3D-REC compared to RAT. There were no comparatively large increases evident when leaves were frozen to lower temperatures. When measurements were performed at 21 °C, a strong increase in respiration rates was recorded at all recovery points for non-acclimated leaves frozen to − 6 °C or − 7 °C. No consistent changes in respiration rates were observed in acclimated leaves measured at 21 °C through the recovery process (Fig. 3d).

### Sugar and proline content after freeze-thaw injury and during recovery

**Fig. 4:**
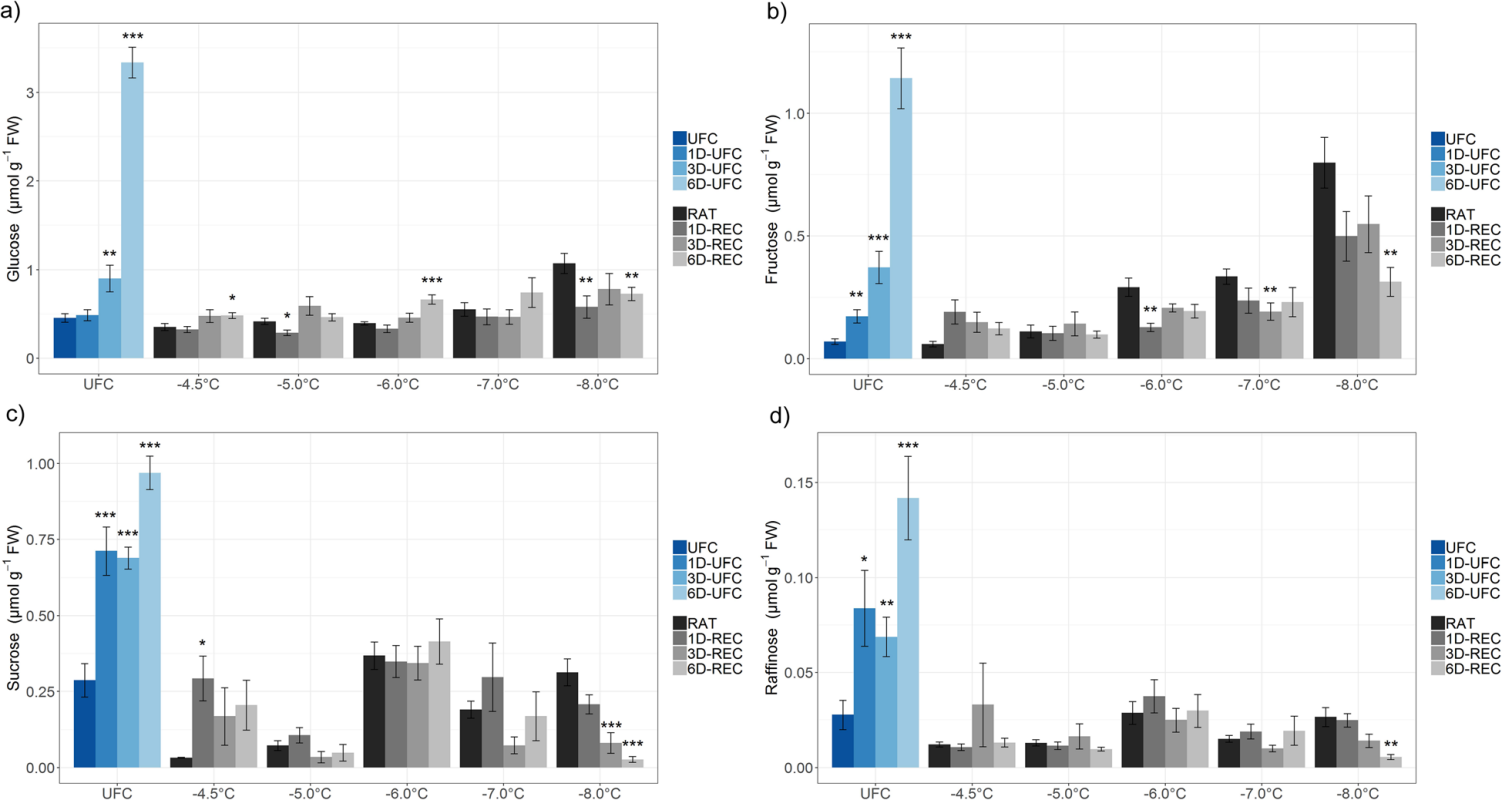
Sugar content in unfrozen Arabidopsis leaves (UFC, 1D-UFC, 3D-UFC, 6D-UFC) and in leaves frozen to different temperatures right after thawing (RAT) or after recovery for 1 day (1D-REC), 3 days (3D-REC) or 6 days (6D-REC) at 4 °C for non-acclimated plants. **a** glucose, **b** fructose, **c** sucrose **d** raffinose. Please note the different scales of the y-axes. Data are the mean with standard errors for five replicates from one experiment except for the non-acclimated UFC and non-acclimated samples at − 6 °C and − 8 °C with three experiments measured. Significance levels between values measured at UFC/RAT and at the subsequent time points determined by Student’s t-test are indicated as: ***, *p* < 0.001, **, *p* < 0.01, *, *p* < 0.05

**Fig. 5:**
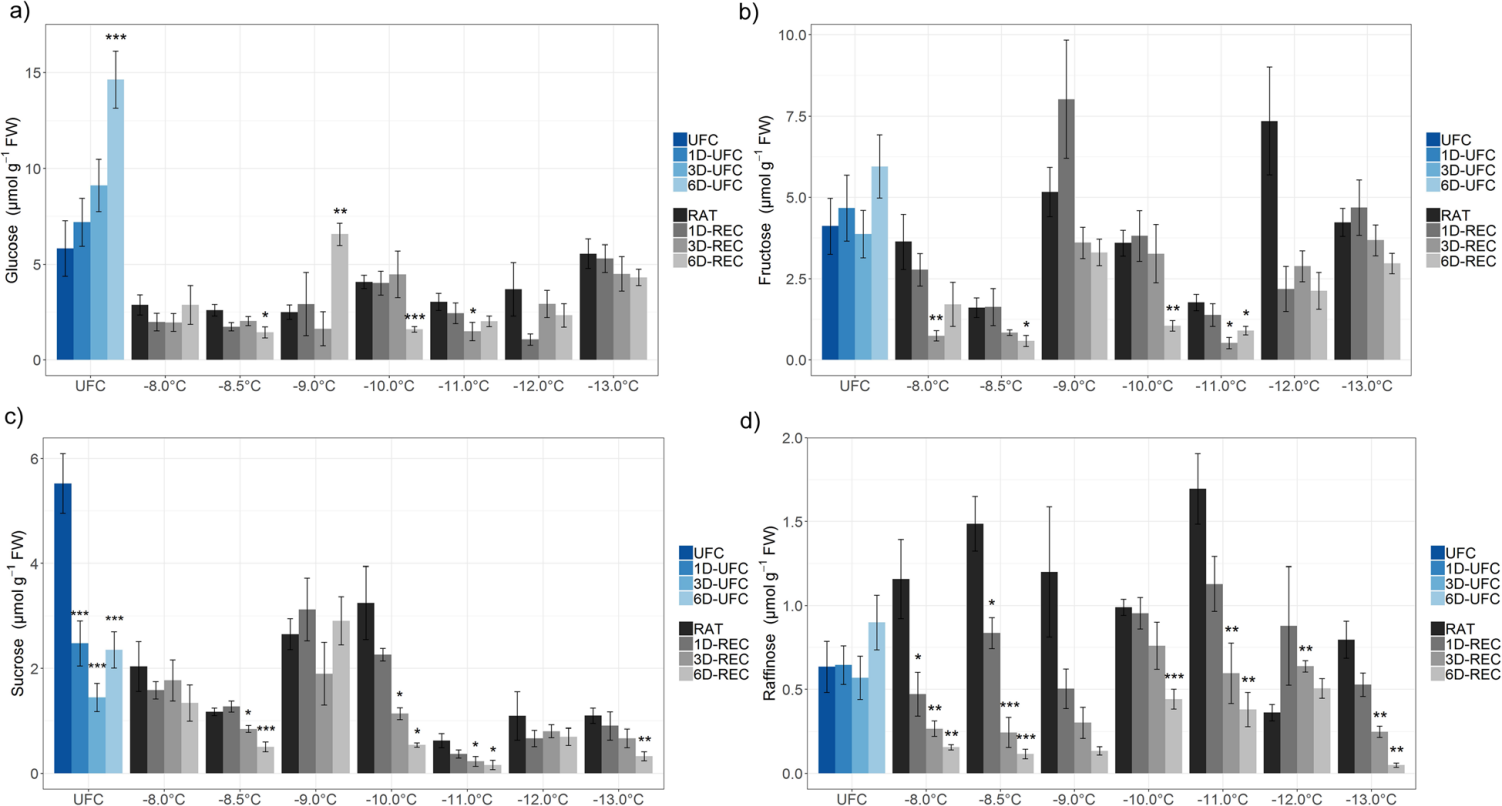
Sugar content in unfrozen Arabidopsis leaves (UFC, 1D-UFC, 3D-UFC, 6D-UFC) and in leaves frozen to different temperatures right after thawing (RAT) or after recovery for 1 day (1D-REC), 3 days (3D-REC) or 6 days (6D-REC) at 4 °C for cold acclimated plants. **a** glucose, **b** fructose, **c** sucrose **d** raffinose. Please note the different scaling of the y-axes. Data are the mean with standard errors for five replicates from one experiment. Significance levels between values measured at UFC/RAT and at the subsequent time points determined by Student’s t-test are indicated as: ***, *p* < 0.001, **, *p* < 0.01, *, *p* < 0.05

**Fig. 6:**
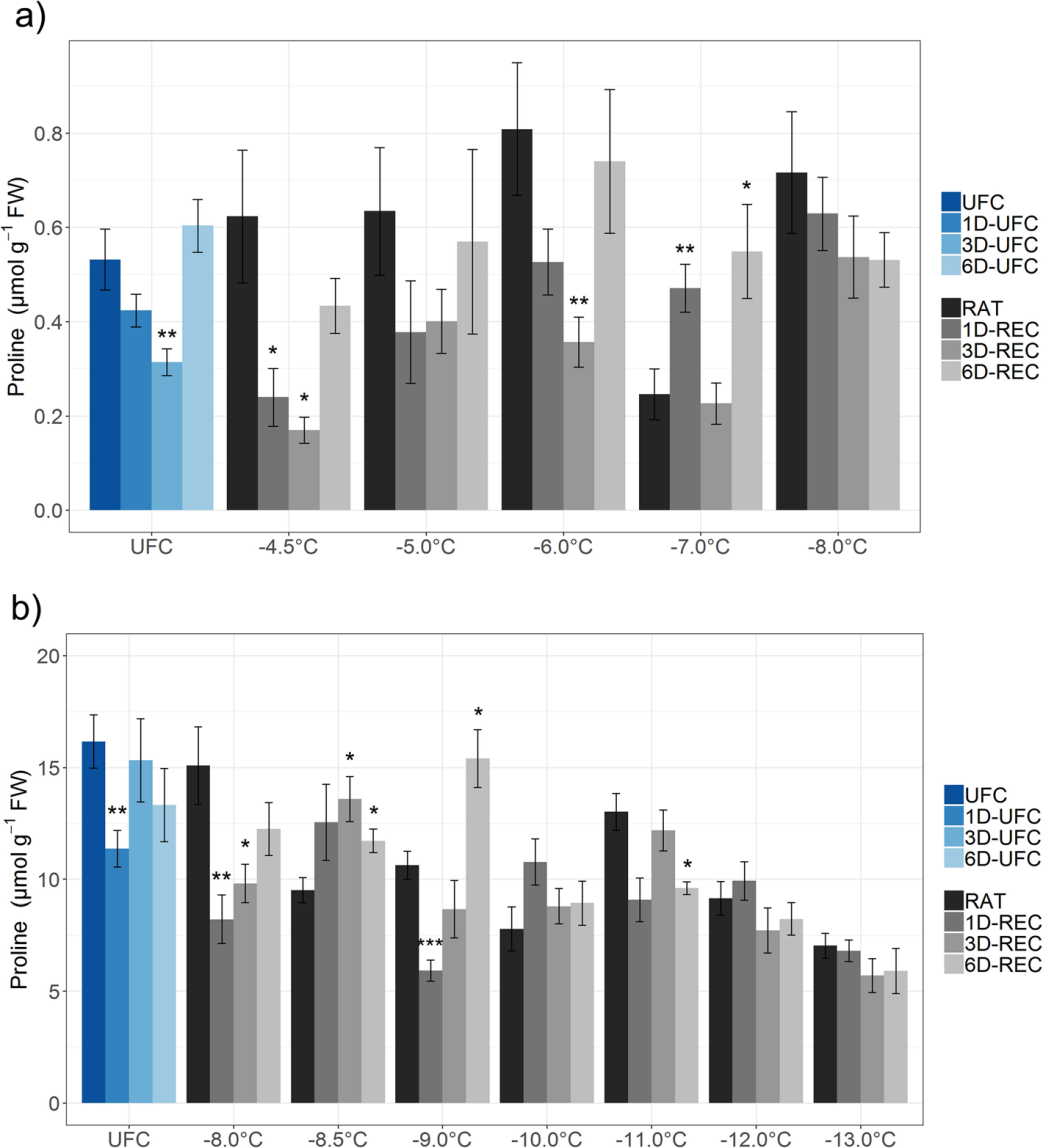
Proline content in unfrozen Arabidopsis leaves (UFC, 1D-UFC, 3D-UFC, 6D-UFC) and in leaves frozen to different temperatures right after thawing (RAT) or after recovery for 1 day (1D-REC), 3 days (3D-REC) or 6 days (6D-REC) at 4 °C for **a** non-acclimated plants and **b** cold acclimated plants. Please note the different scales of the y-axes. Shown is the mean with standard errors for five replicates from one experiment except for the non-acclimated UFC and non-acclimated samples at − 6 °C and − 8 °C with three experiments measured. Significance levels between values measured at UFC/RAT and at the subsequent time points determined by Student’s t-test are indicated as: ***, *p* < 0.001, **, *p* < 0.01, *, *p* < 0.05

Glucose, fructose, sucrose, and raffinose (Figs. 4 and 5) and the amino acid proline (Fig. 6) were measured in leaf samples frozen to temperatures from − 4.5 °C to − 8.0 °C for non-acclimated and from − 8.0 °C to − 13.0 °C for cold acclimated plants. UFC samples from non-acclimated plants showed strong 7.3, 16.4, 3.4 and 5.1 fold increases for glucose, fructose, sucrose and raffinose, respectively, at 6D-UFC compared to UFC conditions (Fig. 4) caused by the experimental design with recovery taking place at 4 °C. Proline content, on the other hand, showed no significant difference between UFC and 6D-UFC in the same samples (Fig. 6a). Cold acclimation for 7 d resulted in a massive accumulation of all sugars (Fig. 5) and proline (Fig. 6), in agreement with many earlier reports [2, 30]. In cold acclimated UFC leaves, changes were more variable than in non-acclimated leaves, with a significant increase in glucose, a significant decrease in sucrose, and no significant changes in fructose, raffinose and proline content between UFC and 6D-UFC (Figs. 5 and 6b).

When leaves from non-acclimated plants were subjected to a freeze-thaw cycle, the levels of glucose (only at − 8 °C) and fructose (at − 6 °C, − 7 °C and − 8 °C) at RAT increased compared to the corresponding value under control conditions. During recovery, no consistent further increases were observed. In fact, at − 8 °C (i.e. below the LT_50_) sugar levels significantly decreased over time (Fig. 4). In leaves from cold acclimated plants frozen to temperatures from − 8 °C to − 13 °C, glucose, and sucrose levels were mostly lower than under control conditions, while fructose levels were similar. Only raffinose content was consistently higher in frozen-thawed leaves at RAT than in the UFC. Recovery had no consistent influence on glucose levels at any freezing temperature, while fructose, sucrose and raffinose levels decreased at most freezing temperatures and recovery time points (Fig. 5). In particular raffinose decreased strongly during recovery.

In non-acclimated leaves proline levels decreased at 1D-UFC and 3D-UFC and at 1D-REC and 3D-REC in samples frozen to − 4.5 °C, − 5.0 °C and − 6 °C, although the decreases were not significant at all temperatures and time points (Fig. 6a). Quite strikingly, proline increased again at 6D-REC and returned to similar levels as at RAT. In leaves frozen to − 8 °C, proline content showed a decreasing tendency over time, that was, however, not significant. In leaves from cold acclimated plants, proline content decreased gradually at RAT with decreasing freezing temperatures and reached 43% of the control value at − 13 °C (Fig. 6b). The pattern of changes in proline during recovery was similar to non-acclimated conditions with an initial decrease and a later increase under control conditions and after freezing to − 8.0 °C and − 9.0 °C. When the leaves were frozen to temperatures between − 10 °C and − 13 °C proline levels did not show consistent changes during 6D-REC.

### Expression of genes encoding proteins related to recovery from freeze-thaw injury

Genes related to the recovery process after a freeze-thaw cycle were selected from a proteomic study in onion [13]. Orthologs of 41 onion genes were identified in Arabidopsis and included genes encoding 12 proteins involved in cell wall remodelling, proteins involved in ion and water homeostasis (e.g. three aquaporins), and in ROS scavenging (e.g. four glutathione S-transferases) (Additional file 1: Table S1). In addition, we included five cold-responsive genes (*COR6.6, COR15A, COR47, COR78, Galactinol synthase 3 - GOLS3*) that we have used previously to characterize transcriptional responses of Arabidopsis to cold acclimation [28] and deacclimation [23]. Furthermore some related genes to the ones identified from the onion study, e.g. genes encoding 14–3-3 proteins (*GRF5–8*) were included. When the expression level of a specific gene was very low at all conditions this gene was excluded from the analysis (e.g. annexin 7 – ANNAT7). cDNAs of leaves from non-acclimated plants frozen to − 5 °C, − 6 °C, − 7 °C, and − 8 °C, and from cold acclimated plants frozen to − 9 °C, − 10 °C, − 11 °C, − 12 °C, and − 13 °C from all recovery time points (RAT, 1D-REC, 3D-REC and 6D-REC) and the respective UFCs were analyzed by qRT-PCR (Additional file 3: Table S3).

**Fig. 7:**
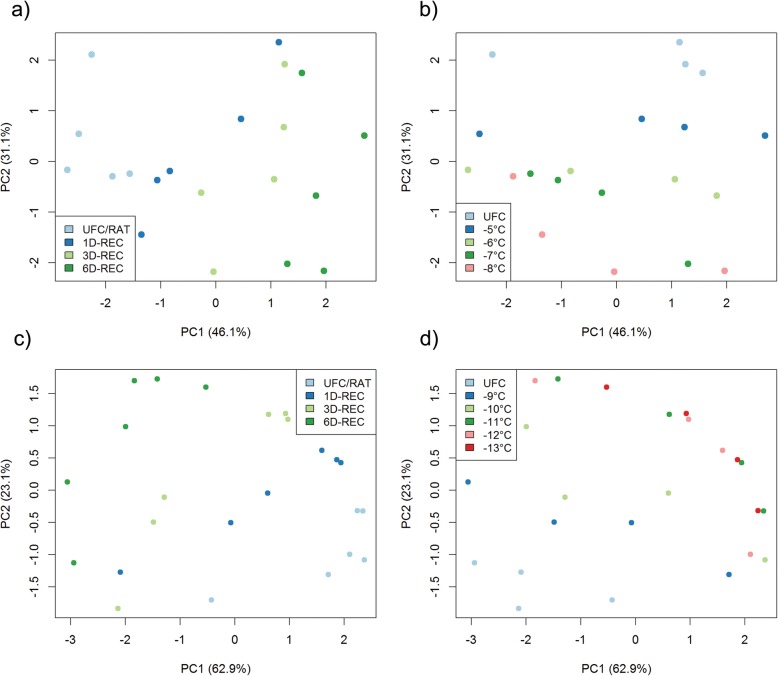
Probabilistic Principal Component Analysis (PCA) using expression data of 41 genes measured by qRT-PCR in unfrozen Arabidopsis leaves (UFC) and in leaves frozen to different temperatures right after thawing (RAT) or after recovery for 1 day (1D-REC), 3 days (3D-REC) or 6 days (6D-REC) at 4 °C for **a**, **b** non-acclimated plants and **c**, **d** cold acclimated plants. Samples are color coded according to days after recovery (**a**, **c**) or freezing temperature (**b**, **d**)

A principal component analysis (PCA) of all generated data showed that Principal Component 2 (PC2) separated samples from non-acclimated and cold acclimated plants, explaining 31% of the variance in the data set (Additional file 4: Figure S1). To increase resolution among data generated from leaves frozen to different temperatures and recovered for various durations, we performed PCAs separately with the datasets generated from non-acclimated and cold acclimated plants (Fig. 7). Samples were colour coded either for recovery duration (Fig. 7a, c) or temperature (Fig. 7b, d). PC1 clearly separated all different recovery times from each other under both non-acclimated and cold acclimated conditions and explained 46.1% or 62.9% of the total variance in the datasets, respectively (Fig. 7a, c). Samples at the RAT timepoint were separated consecutively further from samples after 1D-REC, 3D-REC and 6D-REC. PC2 separated the samples according to the treatment temperatures and explained 31.1 and 23.1% of the variance under non-acclimated and cold acclimated conditions, respectively (Fig. 7b, d). Under non-acclimated conditions, samples were consecutively grouped according to the different treatment temperatures, while under acclimated conditions UFC samples and samples frozen to − 9 °C and − 10 °C were clearly separated from each other. Samples frozen to − 11 °C, − 12 °C and − 13 °C clustered together, but were separated from samples frozen to milder temperatures.

**Fig. 8:**
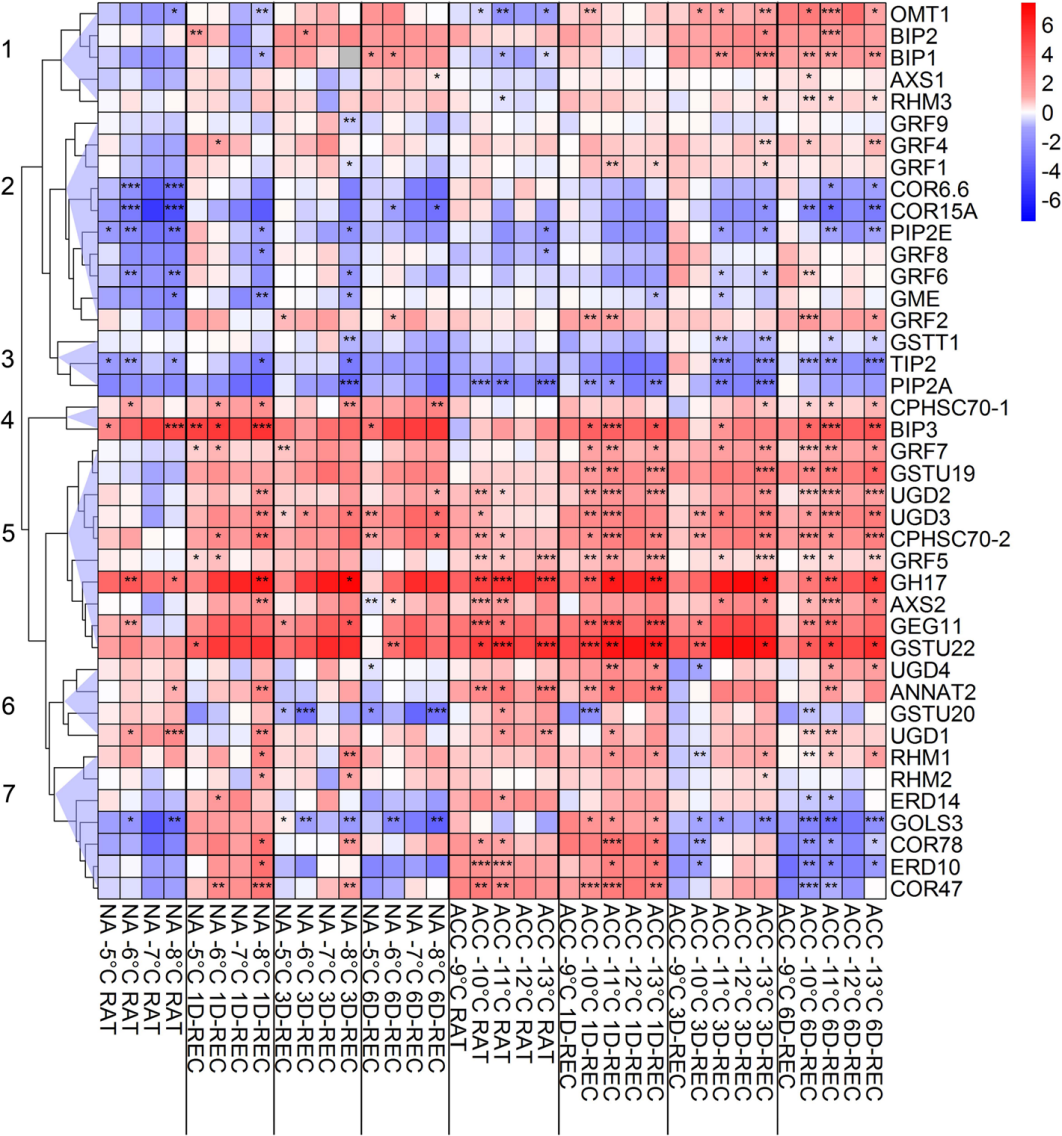
Hierarchical cluster analysis (HCA) of the changes in relative transcript abundance of 41 genes in Arabidopsis leaves frozen to different temperatures right after thawing (RAT) or after recovery for 1 day (1D-REC), 3 days (3D-REC) or 6 days (6D-REC) at 4 °C for non-acclimated plants (left part) and cold acclimated plants (right part). The log_2_ FC in transcript abundance at the different conditions relative to the respective control is color coded with blue indicating reduced and red increased transcript abundance. Seven main clusters are represented as blue triangles on the left. Significance levels of the relative expression to the respective temperature control (2^-∆Ct^) are indicated as: ***, *p* < 0.001, **, *p* < 0.01, *, *p* < 0.05

Figure 8 shows a hierarchical cluster analysis of the changes in transcript abundance (log_2_ fold change) during recovery after freezing to different temperatures relative to the respective control condition. The 41 genes could be grouped into four larger clusters containing five to ten genes (clusters 1, 2, 5, 7) and three smaller clusters containing two to four genes (clusters 3, 4, 6).

**Cluster 1** contains five genes encoding two proteins of the HSP70 family (luminal binding protein 1 and 2, BIP1 and BIP2), a flavonol-3-O-methyltransferase (OMT) and two cell wall related proteins (UDP-D-xylose synthase 1 (AXS1) and rhamnose biosynthetic enzyme 3 (RHM3)). The genes in this cluster were upregulated during recovery with the highest upregulation at 3D-REC and 6D-REC. At RAT conditions, these genes were slightly downregulated in samples of non-acclimated and in selected samples of cold acclimated plants.

Genes in **cluster 2** were mostly downregulated or not significantly changed in expression. Only three genes encoding 14–3-3 like proteins (*GRF1, 2, 4*) showed significant upregulation at some time points/temperatures. In non-acclimated samples, the expression of almost all genes of cluster 2 was decreased at RAT with significant changes for *COR6.6*, *COR15A*, *PIP2E*, *GRF6* and *GME* (*GDP-mannose 3,5-epimerase*). After 1D-REC, 3D-REC or 6D-REC the expression of most of these genes increased slightly to levels of the control in leaves frozen to − 5 °C, − 6 °C or − 7 °C, whereas there was still a significant decrease in the expression of *PIP2E*, *GRF6, GRF8* and *GME* in samples frozen to − 8 °C. A similar pattern was observed in leaves of acclimated plants with the exception that there were almost no significant changes at RAT and a significant decrease of *COR6.6*, *COR15A* and *PIP2E* at 6D-REC.

**Cluster 3** comprises three genes encoding glutathione S-transferase (GST) THETA1 (*GSTT1*), the aquaporins tonoplast intrinsic protein 2 (*TIP2*) and plasma membrane intrinsic protein 2A (*PIP2A*). All three genes showed decreased expression during recovery in both non-acclimated and cold acclimated leaves.

**Cluster 4** only contained two genes encoding heat shock proteins of the HSP70 family (CPHSC 70–1 and BIP3*)*. Both genes were upregulated throughout recovery in non-acclimated and cold acclimated leaves and showed a stable log_2_ FC in comparison to the respective control with recovery time which was independent of the freezing temperature.

The ten genes in **cluster 5** all showed strong up-regulation during recovery, which was, however, more pronounced in cold acclimated than in non-acclimated leaves. The three genes with the highest log_2_ FC with values above five encoded O-glycosyl hydrolase family 17 protein (GH17), glucan endo-1,3-β-glucosidase 11 (GEG11) and GST TAU22 (GSTU22). Only at RAT, in particular in leaves from non-acclimated plants, some of the genes in cluster 5 showed no change in expression or even a mild (non-significant) down regulation.

Of the four genes in **cluster 6**, two (*UDP-glucose dehydrogenase 4* (*UDG4*) and *annexin 2* (*ANNAT2*)) showed increased expression in leaves from cold acclimated plants at RAT and 1D-REC, in particular in leaves frozen to lower temperatures. In contrast, *GSTU20* was mainly down regulated, most strongly in non-acclimated leaves at 3D-REC and 6D-REC. Otherwise, the genes in cluster 6 did not show a coherent expression pattern in response to freezing or during recovery.

Finally, **cluster 7** contained genes that were mainly differentiated in their expression pattern directly after freezing (RAT). At RAT, most of these genes were down regulated in non-acclimated, but up regulated in cold acclimated leaves. At 1D-REC most of these genes were induced, in particular at lower freezing temperatures. This was especially pronounced for the three genes COR78, *ERD10* and *COR47*. At the later recovery time points, the genes in this cluster tended to be less induced (3D-REC) or even strongly repressed (6D-REC) in their expression.

## Discussion

### Membrane repair and restored photosynthesis during recovery

Electrolyte leakage, as a measure of plasma membrane injury, was determined after leaves of non-acclimated and cold acclimated plants were frozen to different temperatures [31]. RAT leakage values increased with decreasing freezing temperatures, showing the direct temperature effect on the extent of leaf damage. Since RAT electrolyte leakage values were determined after over-night thawing, it cannot be excluded that some minor recovery had already taken place at that time. Freezing temperatures for the recovery experiments were chosen so that leaves either displayed recovery or irreversible injury. The latter was reached by freezing to temperatures below the LT_50_, which for non-acclimated *A. thaliana* Col-0 has been established as close to − 6 °C and for cold acclimated plants as close to − 10 °C [28, 32]. We found that freeze-thaw injury was irreversible for leaves of non-acclimated plants frozen to − 7 °C and of cold acclimated plants frozen to − 11 °C, corresponding to leakage values of about 45 and 67%, respectively. During the 6 day recovery period, non-acclimated as well as acclimated leaves that had been frozen to temperatures above the LT_50_ showed reduced electrolyte leakage over time. The largest decrease of electrolyte leakage occurred already at 1D-REC, suggesting that most recovery processes were activated rapidly after the initial freezing stress. This is in agreement with a substantial recovery from freezing damage in spinach leaves and oat crowns after 24 h [22, 27].

Recovery from freezing was additionally detected by measuring the quantum yield of PSII (Fv/Fm) using chlorophyll fluorescence imaging. In both non-acclimated and cold acclimated leaves, Fv/Fm decreased at the RAT time point with decreasing freezing temperatures as could be expected from previous publications [19, 33]. Fv/Fm gradually recovered to values above the RAT value between 1D-REC and 3D-REC in all samples frozen to non-lethal temperatures. Decreasing the temperature by one degree (from − 6 °C to − 7 °C and from − 11 °C to − 12 °C for non-acclimated and cold acclimated leaves, respectively) not only prevented recovery of the photosynthetic efficiency completely, but actually resulted in further reduction in Fv/Fm over time. Presumably, the massive disruption of the thylakoid membrane system that takes place at these low temperatures [34] leads to further degradation of the photosynthetic machinery after thawing.

Fv/Fm did not reach the values obtained for UFC leaves even at 6D-REC except for non-acclimated samples when frozen to − 5 °C, suggesting that full repair of the photosynthetic machinery was not possible in Arabidopsis even after a relatively mild freezing stress, while in spinach leaves photosynthesis was fully recovered at 6D-REC [22]. Similarly, photosynthetic capacity recovered in Scots pine needles to prefrost levels within a few days [35], while full recovery of photosynthetic activity of Norway spruce needles after a frost event was only reached after 60 days [36].

Moreover, the degree of recovery depended on the freezing temperature, indicating that the freezing temperature not only affected the initial damage, but also the extent of possible repair. Similarly, a rapid reactivation of photosynthesis after a 1 week warm spell was described for lingonberry (*Vaccinium vitis-idaea*) during different phases of winter whereas a prior freezing treatment retarded the recovery significantly [37].

### Respiration rate increased during recovery from freezing

We measured respiration rates at two different temperatures, namely the temperature at which leaves were kept for repair (4 °C) and in addition at 21 °C. As expected, respiration rates were three- to fourfold higher in UFC leaves at 21 °C compared to 4 °C. In leaves frozen to non-lethal temperatures, the initial respiration rate at RAT was generally higher in cold acclimated compared to non-acclimated leaves, independent of the measurement temperature. Cold-adapted plants have an increased number of mitochondria in their cells [38], resulting in a more active respiratory machinery after cold acclimation [39]. Decreasing temperatures additionally cause an accumulation of soluble sugars and starch [30]. Thus, an increased availability of substrate [40] could also contribute to an increase in respiration rates in cold acclimated leaves. Functionally, the increased respiration rates during recovery may be required to provide energy for biosynthetic and repair processes [41]. In addition, respiration may also be involved in ROS scavenging. Two terminal oxidases, cytochrome C oxidase (COX) and alternative oxidase (AOX) are involved in the respiratory pathway. AOX as non-energy conserving terminal oxidase greatly reduces the ATP yield during respiration because no proton-pumping across membranes is required due to bypassing of proton-pumping complex III and IV [42]. The *AOX* gene is upregulated during cold stress [43] which might lead to a reciprocal reduction of the COX pathway. Furthermore, silencing of *AOX* in *Arabidopsis* causes accumulation of ROS [44]. Hence, it has been suggested that AOX acts as a ROS scavenger, further supported by an activation of the AOX respiratory pathway during recovery from injury [44].

The recovery of non-acclimated samples was performed at a temperature of 4 °C, so the increase of the respiration rate in UFCs and samples frozen to − 6 °C and − 7 °C could also, at least partially, reflect the adaptation of the respiratory machinery to cold conditions in addition to the recovery process. Further, our data show that respiratory activity was still detectable in non-acclimated leaves frozen to − 8 °C at 6D-REC, when Fv/Fm was no longer detectable. This indicates that respiration is more robust under freezing conditions than photosynthesis, which may be functionally significant to ensure effective repair of partially damaged cells. Differential tolerance by various cellular processes to freeze-thaw treatment has previously been reported in potato leaves where photosynthesis was found to be much more sensitive than respiration [45].

### Sugars and proline may serve as energy and nitrogen sources during recovery

The initial sugar and proline content in unfrozen Arabidopsis control leaves was around ten times higher in acclimated than non-acclimated leaves, as reported previously [28, 46]. Increases in sucrose, raffinose, and other cryoprotectants occur in parallel to a starch breakdown at the beginning of cold acclimation and transcriptional activation of genes encoding glycolytic enzymes supports the increasing demand for carbon skeletons for cryoprotectants [30].

Soluble sugars and some amino acids such as proline stabilize both membranes and proteins during freezing (e.g. [47–49]). Moreover, both sugars [50, 51] and proline [52, 53] can act as ROS scavengers and may thereby also contribute to plant freezing tolerance. Obviously, the high amounts of sugars and proline that are accumulated during cold acclimation could also serve as carbon and nitrogen sources for repair processes after a freeze-thaw cycle, and the sugars could additionally provide the energy for repair through respiration, as discussed above.

UFC leaves from non-acclimated plants showed a continuous increase in the content of all four measured sugars over time. Since the leaves were incubated at 4 °C in the dark overnight and then at 4 °C with a 16 h day length during 6 days, we assume that this is due to a cold acclimation response. This conclusion is supported by an upregulation in the expression of the known cold-induced genes *GolS3*, *COR6.6*, *COR15A*, *COR78* and *COR47* in these samples with expression values higher or similar to RAT (0 day control) values from cold acclimated plants (Additional file 3: Table S3). Interestingly, proline did not significantly accumulate in non-acclimated leaves over 6 days under the same conditions. A delay in proline compared to sugar accumulation of approximately 1 day has been reported previously at 1 °C [54]. The longer delay observed in our experiments may be due to the fact that we investigated detached leaves while the earlier study was conducted with whole plants.

After freezing to sub-lethal temperatures, non-acclimated leaves showed very little change in sugar content during recovery, which may be due to increased sugar consumption by respiration, but may in addition also be related to damage to the photosynthetic machinery and reduced gluconeogenesis. The reduced sugar content in leaves frozen to the lowest temperature, where no repair was observed, was most likely related to a progressive breakdown of sub-cellular compartmentation under these conditions [55].

In cold acclimated leaves sugars, except for glucose, were mostly decreased during recovery, probably caused by a higher demand for carbohydrates for respiration. Similarly, sucrose and fructose were strongly decreased after a 14 day recovery period after freezing in overwintering crown tissue of *Avena sativa* [27].

Most of the frozen-thawed leaves from non-acclimated plants displayed a decrease in proline content during the first 3 days of recovery, while it increased again 6D-REC. A similar pattern was found in some of the cold acclimated samples, but with an increase in proline content already by 3D-REC. Proline may have been degraded during the early recovery phase to serve as nitrogen source for the repair of freezing injury. Once repair was largely finished, the demand for proline may have been reduced and cold-induced synthesis predominated again, leading to an increase in proline content. In cold acclimated leaves, more proline was available at the start of the repair process and therefore net proline accumulation was observed earlier despite its degradation. In overwintering, frozen-thawed crown tissue of *Avena sativa*, proline, 5-oxoproline and arginine content increased over 14 days and this increase was significantly correlated with recovery [27]. At the lowest freezing temperatures, however, proline content slightly, but consistently decreased over time, in agreement with a lack of repair activity.

### Changes in gene expression after freezing and during recovery

Immediately after freezing and during post-thaw recovery many changes in transcript abundance were observed, depending in a complex manner on the gene, the duration of recovery, the freezing temperature and the cold acclimation treatment. Cold-responsive genes (*COR6.6*, *COR15A, GOLS3*, *COR78*, *COR47*) were immediately downregulated after freezing in non-acclimated leaves, and were either unchanged or upregulated in cold acclimated leaves. In both cases they showed an upregulation or no change at 1D-REC and a consistent downregulation at 3D-REC and 6D-REC, indicating a decreasing role of cold-responsive genes during recovery. Together with the reduction in osmolyte content this suggests a general rerouting of metabolism from maintaining freezing tolerance towards repair.

The three investigated aquaporin genes (*PIP2A*, *PIP2E*, *TIP2*) showed reduced transcript abundance directly after freezing and throughout the recovery period. The expression of aquaporin genes *PIP2.1* and *TIP* was also downregulated in spinach leaves injured by mild freezing, but *PIP2.1* expression was partially restored at 3D-REC and 6D-REC, while *TIP* expression increased 1.5-fold over the control level at 6D-REC [22].

Cell wall remodeling is an important process during recovery from freezing [16] and we have investigated the expression changes of eight genes encoding enzymes involved in this process. Four *UGD* genes were induced over the recovery period in both non-acclimated and cold acclimated leaves. The corresponding enzymes are involved in the biosynthesis of UDP-glucuronic acid for the synthesis of pectins and hemicelluloses [56]. A distinct role of the different UGD proteins in carbon partitioning between cell wall synthesis and sucrose synthesis was suggested [56]. An overexpression of an *UGD* gene from *Larix gmelinii* in Arabidopsis resulted in increased levels of sugars and hemicellulose combined with enhanced growth and freezing tolerance [57]. In addition, *AXS2*, which encodes an enzyme that converts UDP-D-glucuronate to a mixture of UDP-D-apiose and UDP-D-xylose, was also induced during recovery. D-apiose is found in rhamnogalacturonan II, apiogalacturonan, and several apioglycosides. An UDP-apiose/xylose synthase was among the most highly induced proteins during recovery from freezing in onion [13].

*GH17* and *GEG11* with induced expression during the whole recovery phase encode proteins involved in the hydrolysis of β-1,3-glucoside linkages [58]. An increase of β-glucanase activity in the cold has also been described in winter rye [59]. In addition to their enzymatic function that relates them to cell wall remodeling, β-1,3-glucanases also have ice binding and ice recrystallization inhibition activity [59] and are able to directly stabilize plant membranes during freezing [60].

The massive upregulation of two *GST* genes (*GSTU19* and *GSTU22*) during recovery may be taken as evidence for the need of additional ROS scavenging under these conditions. Indeed, it has been shown previously that in spinach leaves both superoxide and H_2_O_2_ are accumulated directly after thawing from a sub-lethal freezing temperature and that these ROS are rapidly reduced during recovery [22]. At the same time the activity of the antioxidant enzymes catalase, ascorbate peroxidase and superoxide dismutase is increased. Antioxidant enzymes including several GSTs increased in onion during repair of freeze-thaw-injury [13]. Enzymes with antioxidative function or their transcripts also showed higher abundance during recovery from drought stress [61].

GSTs are involved in cellular protection against oxidative stress and in particular in regulation of the H_2_O_2_ balance [62]. They reduce oxidative stress by catalyzing the formation of a disulfide bridge between two glutathione molecules, thus forming glutathione disulfide. The electrons released in this reaction are then available for the reduction of radicals [62]. GSTs can in addition bind to a variety of exogenous and endogenous ligands which might be damaging to the cell [63]. GSTU19, for instance, catalyzes the glutathionylation of 12-oxophytodienoate (OPDA), a precursor of jasmonic acid. The resulting OPDA-GSH conjugate is then stored in the vacuole [64]. GSTs can also function as carrier proteins for the phytohormones auxin and cytokinin and bind fragments of chlorophyll [65]. Whether any of these functions that are not directly related to ROS scavenging play a role in repair remains to be investigated.

Freezing can cause protein aggregation induced by cell contraction and concentration of the cytoplasm [13] and soluble enzymes may be inactivated during an in vivo freeze-thaw cycle [20]. During repair, such proteins need to be either proteolytically removed or renatured by chaperones. In agreement with this proposition, several HSP genes and proteins, respectively, were induced during recovery in onion [13] and spinach [22]. HSPs are involved in proteostasis, the maintenance of the cellular proteome. We determined the expression of five genes encoding HSP70 family proteins during the recovery process. All five genes (*BIP1, BIP2*, *BIP3*, *CPHSC70–1* and *CPHSC70–2*) were highly up regulated during recovery. BIP proteins are HSP70 proteins of the ER and are able to bind client proteins to prevent their aggregation [66]. BIPs function as chaperones in processes such as protein folding, protein translocation and quality control [67]. They also act as master regulators of Arabidopsis stress responses [68] and play a putative role in the assembly of multimeric protein complexes in the ER [66].

Two other *HSP70* genes (*CPHSC70–1* and *CPHSC70–2*) were also upregulated throughout recovery. They are nuclear encoded and transported into chloroplasts [69]. A knock-out mutant of *CPHSC70–1* is retarded in growth and has abnormal leaves, in contrast to a *CPHSC70–2* mutant that shows no phenotypic effect under normal growth conditions [69]. However, double mutants of both genes are lethal. Artificial microRNA approaches showed that these genes are essential for a normal plastid structure [70].

Two genes encoding 14–3-3 proteins were up regulated during recovery, the general regulatory factors *GRF5* and *GRF7*. This is in agreement with proteomic data in onion, showing increased abundance of two 14–3-3 proteins during recovery [13]. Both Arabidopsis proteins are phosphorylated by cold-responsive protein kinase 1 (CRPK1), inducing their translocation to the nucleus where they interact with CBF transcription factors and promote their degradation [71]. Knockout of *GRF7* results in enhanced and overexpression in reduced freezing tolerance. GRF5 is in addition involved in cytoskeleton organization by activating the microtubule-associated protein Endosperm defective 1 (EDE1), which is essential for cell division and microtubule organization during early stages of mitosis [72]. Their role as negative regulators of the CBF regulon is in agreement with our hypothesis that plants actively shift their metabolism from maintaining or inducing their freezing tolerance in favor of repair processes.

## Conclusions

In the present paper we have addressed research questions central to understanding repair processes in leaves after freezing, highlighting its dependence on the severity of damage, as well as its metabolic and transcriptional basis. Leaves of non-acclimated and cold acclimated Arabidopsis plants showed a fast and continuous recovery, measured as membrane repair accompanied by a restored photosynthesis, only after freezing to sub-lethal but not to lethal temperatures. Increased respiration rates and unchanged or declining levels of compatible solutes, such as sugars and proline, were suggested to act as energy, carbon and nitrogen sources for the repair process. Higher expression of genes encoding proteins important for cell wall remodeling, ROS scavenging and maintenance of the cellular proteome during recovery occurred in parallel with a down regulation of cold-responsive genes, especially in cold acclimated leaves. Transcriptional regulation, e.g. by 14–3-3 proteins, was suggested to be involved in post-thaw recovery. This work contributes to a deeper understanding of recovery processes after sub-lethal freeze-thaw injury, which gains increasing importance due to global climate change and highly fluctuating temperatures that have an increasing effect on plant survival.

## Methods

### Plant material

*Arabidopsis thaliana* accession Columbia-0 (Col-0) originally ordered from NASC (Nottingham Arabidopsis Stock Center, United Kingdom) and propagated for several years at the MPI-MP (Potsdam, Germany) was used in all experiments. After sowing, plants were grown on soil in a climate chamber at 20 °C day-time temperature and 6 °C night-time temperature in a 12 h day light-dark cycle with a light intensity of 180 μE m^− 2^ s^− 1^ and humidity of 70% (day) and 80% (night) for 1 week. The plants were then grown at short day conditions (8 h day length, 180 μE m^− 2^ s^− 1^, humidity of 60/75% day/night) with temperatures of 20 °C and 16 °C (day/night) for 3 weeks. Then plants were transferred to long day conditions (16 h light) at 20 °C day and 18 °C night temperature and light intensity of 200 μE m^− 2^ s^− 1^ for 2 weeks. After these 6 weeks of growth, non-acclimated plants were used in experiments. For cold acclimation, plants were kept for an additional week in a growth chamber at 4 °C and a 16 h day length (90 μE m^− 2^ s^− 1^ light intensity, 70–80% humidity) [28].

For recovery, detached leaves in glass tubes were transferred back after freezing and thawing to the growth chamber used for cold acclimation for 1, 3 or 6 days. Leaves in tubes were kept out of direct light at 15 μE m^− 2^ s^− 1^ light intensity to prevent damage caused by photooxidative processes. Measurements were performed on the leaves right after thawing (RAT) and after 1, 3 or 6 days of recovery (REC) at 8:30 am (2 ½ h after lights on). In addition, samples were taken for proline, sugar and transcript analysis and were stored at − 80 °C.

### Freezing and electrolyte leakage

Freezing experiments were performed according to the method previously described [31]. Two to three leaves, collected from individual plants, were transferred to glass tubes containing 300 μl of water. Control tubes were kept on ice throughout the experiment (unfrozen control – UFC, 1D-UFC, 3D-UFC, 6D-UFC), whereas all other tubes were moved to a programmable silicon oil bath CC130 (Huber, Offenburg, Germany) at a temperature of − 1 °C. After half an hour, ice was added to initiate ice crystallization in the leaves. After an additional 30 min, the oil bath was set to a cooling rate of 4 °C per h. Freezing temperatures above or below the LT_50_, the temperature where 50% of electrolytes leak out of the cell, of non-acclimated (LT_50_ NA) (− 3.0 °C, − 3.5 °C, − 4.5 °C, − 5 °C or − 7 °C) or cold acclimated plants (LT_50_ ACC) (− 8.0 °C, − 8.5 °C or − 11 °C) were chosen according to previously published results (LT_50_ NA: -5.34 °C NA, LT_50_ACC: -9.68 °C) [28] (Table 1). Samples were removed from the bath at appropriate temperatures and left on ice to thaw overnight. Leaves were covered with 7 ml of water and tubes were gently shaken for 24 h at 4 °C before measuring electrolytes at RAT conditions. For samples after 1D-REC, 3D-REC or 6D-REC at 4 °C, water was added at the respective day and electrolyte leakage was measured as above. A detailed sampling scheme is shown in Additional file 5: Figure S2.

For electrolyte leakage measurements, 4.5 ml deionized water was mixed with 1 ml of the water used to shake the leaves in and conductivity was measured. Afterwards tubes containing the plant material and the remaining water were boiled for 30 min and left to cool to room temperature before a second measurement was carried out to obtain the 100% electrolyte leakage value for each sample. The ratio between the two values was calculated and control values were subtracted to obtain the final electrolyte leakage values. For the characterization of recovery processes one experiment was performed with four biological replicates derived from different sets of plants for each freezing temperature and condition.

For all other analyses leaf samples were collected after freezing to lower temperatures than for the electrolyte leakage experiments, since the 24 h-incubation in water, which leads to additional damage to the leaf tissue, was not necessary [19]. For chlorophyll fluorescence imaging leaves were frozen to − 5 °C, − 6 °C and − 7 °C to elicit non-lethal and − 8 °C to elicit lethal damage to non-acclimated plants, or − 9 °C, − 10 °C and − 11 °C (non-lethal) and − 12 °C and − 13 °C (lethal) using cold acclimated plants (Table 1). Since respiration measurements were done at two different temperatures (4 °C and 21 °C) only a limited number of samples could be processed and only samples frozen to − 6 °C and − 7 °C for non-lethal and − 8 °C for lethal damage using non-acclimated plants, or − 8 °C and − 9 °C for non-lethal and − 12 °C for lethal damage using cold acclimated plants were measured. For sugar and proline analysis, additional samples were analyzed that have been frozen to − 4.5 °C for non-acclimated plants or − 8 °C and − 8.5 °C for cold acclimated plants to generate an overlap with freezing temperatures used for electrolyte leakage measurements. Samples were always taken 1 day after freezing (RAT) and 1D-REC, 3D-REC and 6D-REC.

### Chlorophyll fluorescence imaging

Detached *Arabidopsis* leaves were frozen and thawed as described above. Samples were dark adapted for at least 30 min before measuring chlorophyll fluorescence using an IPAM (imaging pulsed-amplitude modulated) fluorometer (Walz, Germany) [31]. The false color images of two to three leaves from five replicates each per freezing temperature and condition were taken (*n* = 10–15) per experiment and data processed using image processing software provided with the instrument (ImagingWinGigE_246r, Walz). The whole leaf area was integrated to calculate a mean Fv/Fm reflecting the potential quantum use efficiency of photosystem II [73]. Three independent experiments were performed including samples frozen to − 6 °C, − 8 °C, − 9 °C, − 10 °C, two experiments for samples frozen to − 13 °C and one experiment including samples frozen to − 5 °C, − 7 °C, − 11 °C and − 12 °C.

### Respiration

Respiration measurements were performed as described previously [74]. Leaves were dark adapted for 30 min and then leaf slices were cut from two leaves per replicate in dim light and placed into 1.5 ml vials (Supelco Analytical, Bellefonte, CA) filled with 25 mM imidazole buffer (pH 6.5). Air bubbles were removed using an ultra-sonic water bath Sonorex RK100 (Bandelin, Berlin, Germany). Vials were topped up with buffer and closed with screw caps containing a silicone inlet (Chromacol, Welwyn Garden City, England) without trapping air bubbles. Vials were incubated in darkness at either 21 °C or 4 °C. Oxygen consumption was measured in the dark at three consecutive time points with a Microx TX2 system and a needle mounted optical oxygen microsensor (NTH Pst 1-LS-TS-NS 40 × 0, PreSens, Regensburg, Germany) using the program TX2 OxyView V4.2 (PreSens). The sensor was calibrated against ambient air (100% oxygen saturation) and a 1% (w/v) sodium sulfite solution (0% oxygen saturation). After measurement, leaf slices were removed from the vials, shortly dried on paper and weighed. Respiration rates were calculated as nmol O_2_ min^− 1^ mg^− 1^ FW. Experiments were performed with five replicates per freezing temperature and recovery condition.

### Sugar and proline measurements

Sugars and proline were quantified from detached leaves after the different treatments as previously described [28]. Samples were frozen in liquid nitrogen and homogenized using a ball mill ‘Retsch MM 200’ (Retsch, Haan, Germany). Soluble sugars were extracted with 80% ethanol from 50 mg of frozen leaf material, desalted and quantified by high performance anion exchange chromatography using a CarboPac PA-100 column on an ICS3000 chromatography system (Dionex, Sunnyvale, CA, USA). Proline was quantified photometrically from the same ethanol extracts using the acidic ninhydrin method [28, 75]. Samples from one experiment were measured with five replicates per freezing temperature at RAT and 1D-REC, 3D-REC and 6D-REC except for the non-acclimated UFC, − 6 °C and − 8 °C with three experiments measured.

### qRT-PCR analysis of gene expression

Total RNA was extracted from leaf material pooled from five replicate samples per freezing temperature and recovery condition from up to four biological experiments. For the control samples and − 9 °C for cold acclimated samples *n* = 4, for − 6 °C, − 8 °C, − 13 °C and for the non-acclimated control *n* = 3. For − 5 °C and − 11 °C *n* = 2 and for − 7 °C, − 9 °C, − 12 °C *n* = 1. RNA extraction, DNAse treatment and RNA quality control using forward and reverse intron specific primers (MAF5 primer, AT5G65080) was performed according to a previously described method [76]. First strand cDNA synthesis and cDNA quality controls using forward and reverse 3’GADPH and 5’GADPH primers (AT1G13440) and quantitative PCR measurements were performed as published [28]. Reactions with 2.5 μl 2 × SYBR Green Master Mix (Fast Power SYBR Green; Applied Biosystems, Darmstadt, Germany), 0.5 μl cDNA (diluted 5-fold) and 2 μl of 0.5 μM primers were pipetted using an Evolution P3 pipetting robot (PerkinElmer, Zaventem, Belgium) and measured using an ABI PRISM 7900 HT 384-well plate Sequence Detection System (Applied Biosystems).

Genes of interest for the expression analysis were chosen from a proteomic analysis of onion scales comparing UFC, freeze-thaw injured and post-thaw recovered tissues [13]. Orthologs of potential recovery related proteins in onion were identified in Arabidopsis (Additional file 1: Table S1) and respective gene sequences were selected together with some related genes for primer design. Primers were either designed in Primer3 (http://primer3.wi.mit.edu/) [77, 78] or taken from the literature. The specifications of the designed primers were as follows: primer length 20–24 bases, amplicon size 60–150 bp, primer melting temperature 64 ± 3 °C, amplicon melting temperature 75–95 °C, G/C content 45–55%, maximum repetition of a nucleotide 3 and a G/C clamp of 1. Further, primers that hybridize close to the 3′ end of the gene were preferably chosen. Sequences of all primers can be found in Additional file 2: Table S2. Ct values for the genes of interest were normalized by subtracting the mean Ct of the three reference genes, *Actin2*, *EXPRS* and *PDF2* [28], and averages of all biological replicates were determined. Relative expression changes were calculated as log_2_ fold-change compared to the respective control and were visualized in heat maps generated using Pheatmap in R [79]. Hierarchical cluster analysis was performed using Pearson correlation with average linkage. The significance of differences between the conditions was calculated using an unpaired two-sided t-test in Excel.

### Further data analysis methods

Principle Components Analysis (PCA) was performed using ∆Ct values, which were normalized to the median of all samples for each gene and taken to the log10. Probabilistic PCA was performed using the *pcaMethods* package in R [80].

## Supplementary information

**Additional file 1: Table S1.** Orthologs of potential recovery related proteins identified by a proteomic study in onion [13] and identified in Arabidopsis by BLAST search.

**Additional file 2: Table S2.** Sequences of the primers used for qRT-PCR analysis.

**Additional file 3: Table S3.** Relative expression (2^-∆Ct^) of 41 genes in Arabidopsis leaves frozen to different temperatures right after thawing (RAT) or after recovery for 1 day (1D-REC), 3 days (3D-REC) or 6 days (6D-REC) at 4 °C for non-acclimated and cold acclimated plants.

**Additional file 4: Figure S1.** Probabilistic Principal Component Analysis (PCA) using expression data of 41 genes measured by qRT-PCR in Arabidopsis leaves frozen to different temperatures right after thawing or after recovery for 1, 3 or 6 days at 4 °C for non-acclimated (NA) plants (red dots) and cold acclimated (ACC) plants (blue dots). The mean gene expression was divided by the median gene expression across all condition and the log10 was calculated.

**Additional file 5: Figure S2.** Sampling scheme for the recovery of detached leaves from non-acclimated and cold acclimated plants after freezing. RAT – right after thawing, REC – recovery, UFC – unfrozen control, D - day.

## Data Availability

All data generated or analysed during this study are included in this published article and its supplementary information files or are available from the corresponding author on request.

## Abbreviations

ACC: Cold acclimated
AOX: Alternative oxidase
COR: Cold responsive genes
COX: Cytochrome C oxidase
D-REC: Day after recovery
FTST: Freeze-thaw stress tolerance
FW: Fresh weight
GSH: Glutathione
HSP: Heat shock protein
LT_50_: Leakage temperature at which 50% of damage occurs
NA: Non-acclimated
PC: Principal component
PCA: Principal component analysis
RAT: Right after thawing
ROS: Reactive oxygen species
UFC: Unfrozen control
