# Supplementary material for: Repair of sub-lethal freezing damage in leaves of Arabidopsis thaliana
Source: BMC Plant Biol. 2020 Jan 20;20:35. doi: 10.1186/s12870-020-2247-3 (PMC6971927; doi:10.1186/s12870-020-2247-3)
Supplement: Supplementary file 2 — Additional file 2: Table S2. Sequences of the primers used for qRT-PCR analysis. [file 12870_2020_2247_MOESM2_ESM.pdf]

Suppl. Table 2: Sequences of the primers used for qRT-PCR analysis.

Locus ID	Description	Gene ID	Primer Sequence 5'→3'		Reference
AT1G08200	UDP-D-xylose synthase 2	AXS2	FW	TGGCGGATTCCACACTTCACTTC	designed in Primer3
			RV	TCACATAACACAGCAACACGAGC	
AT1G09080	Binding protein 3	BIP3	FW	TTTGTGACCCGGTTATTAAGTCGG	designed in Primer3
			RV	TCATCGTGATCATCTCCATCGTCG	
AT1G09350	Galactinol synthase 3	GOLS3	FW	ACAGGCCAAGAAGGAAATATGG	Nishizawa *et al.* 2008
			RV	GATGGAGCTTTGGCACATTG	
AT1G20440	Cold-regulated 47	COR47	FW	ACAAGCCTAGTGTCATCGAAAAGC	Rhode *et al.* 2004
			RV	TCTTCATCGCTCGAAGAGGAAG	
AT1G20450	Early response to dehydration 10	ERD10	FW	TCTCTGAACCAGAGTCGTTT	Jiang *et al* 2011
			RV	CTTCTTCTCACCGTCTTCAC	
AT1G26570	UDP-glucose dehydrogenase 1	UGD1	FW	AGGACACAGGTGACACGAGAGAG	designed in Primer3
			RV	TCAGCTTGGCCTTGTCTGCAAC	
AT1G32860	Glucan endo-1,3-beta-glucosidase 11	GEG11	FW	AGATCGACGCTGTCTACTCTGC	designed in Primer3
			RV	ATCGTCGTCACCTTTCGATGGC	
AT1G35160	General regulatory factor 4	GRF4	FW	CACCGATCTCTAATGGCGGCAC	designed in Primer3
			RV	TCGTAACGTTCCGCTTGCTCTG	
AT1G53500	Rhamnose Biosynthesis 2	RHM2	FW	CCGAGAATGCTTATGATGCCCGG	designed in Primer3
			RV	CCGTAAATGTCTGGACCGTGTTGC	
AT1G76180	Early response to dehydration 14	ERD14	FW	GTGGAAGAAGCGCATCCAGTGG	designed in Primer3
			RV	ACGGTGGTCTTAGGGTGGTATCC	
AT1G78300	General regulatory factor 2	GRF2	FW	ACTCTAGACTCATCCCTGCCGC	designed in Primer3
			RV	GTATGTTCGGCGGCGTCTTTCC	
AT1G78340	Glutathione S-transferase TAU 22	GSTU22	FW	AGAGAATCTGAGAGACAAGAGCCC	designed in Primer3
			RV	CGGACCAAACCTCGTCGATGTAC	
AT1G78370	Glutathione S-transferase TAU 20	GSTU20	FW	TCGAGTTGGTTCCAAGCCTATGAG	designed in Primer3
			RV	TCGGACTCTCTGACTCGATGC	
AT1G78380	Glutathione S-transferase TAU 19	GSTU19	FW	AAGGGTGAGGAACAAGAGGCAG	designed in Primer3
			RV	ACATAGCCAAAGTCATCGCCAC	
AT1G78570	Rhamnose biosynthesis 1	RHM1	FW	GGAAAGGTCGGTTGGAGGATCG	designed in Primer3
			RV	CCCAGTCACACCAGCGGAATTG	
AT2G27860	UDP-D-xylose synthase 1	AXS1	FW	TGGGCACATCTTCAACGTAGGC	designed in Primer3
			RV	TGGGCTCTCAATGGCTCCTTCTC	
AT2G39010	Plasma membrane intrinsic protein 2e	PIP2E	FW	GTCGGTCCATTTGTGGGTGCAG	designed in Primer3
			RV	TGAAGCTGGCTCCTGACTGACC	
AT2G42540	Cold-regulated 15A	COR15A	FW	AACGAGGCCACAAAGAAAGC	Rhode *et al.* 2004
			RV	CAGCTTCTTTACCCAATGTATCTGC	
AT2G42590	General regulatory factor 9	GRF9	FW	TGCTCATAAGACGAATGGTTCTGC	designed in Primer3
			RV	CATATCACTCTGCATCGTCTCCAC	
AT3G02520	General regulatory factor 7	GRF7	FW	AATGATGAGGCGGGCGGTGATG	designed in Primer3
			RV	TGCCCTGTCTCAGCTGGTTTCC	
AT3G04010	O-glycosyl hydrolases family 17 protein	GH17	FW	ACCGTTCTTGACAGCGTACAACG	designed in Primer3
			RV	TCGGCGTTTAAAGGAACGGTGG	
AT3G14790	Rhamnose biosynthesis 3	RHM3	FW	CAAGTCATGTCTTCAACGCCGC	designed in Primer3
			RV	TCTGCAAGAGTCAAAGTTCCAGC	
AT3G26520	Tonoplast intrinsic protein 2	TIP2	FW	TGCCGGAGTCGGATCATTAAACG	designed in Primer3
			RV	TGAAACCTATGGCGATTGGTGCG	
AT3G29360	UDP-glucose dehydrogenase 2	UGD2	FW	ATTTGCAGCCAATGAGCCCAAC	designed in Primer3
			RV	CGTATGCGTCCCAAGTAACGGTC	
AT3G53420	Plasma membrane intrinsic protein 2a	PIP2A	FW	TACAGCACAGGGACCGGTCTAG	designed in Primer3
			RV	ACGTGGGAGTCTCTGGCACTAC	
AT4G09000	General regulatory factor 1	GRF1	FW	CTGACGAGCAACAATCCTAAGTCG	designed in Primer3
			RV	AGACTGCCTTAGAGAGCTTGGG	

AT4G24280	Chloroplast heat shock protein 70-1	CPHSC70-1	FW	AGGAACTCGTGAGGAAGGTGAC	designed in Primer3
			RV	CTCCAGCAAGAACACCAGCCTG	
AT5G10450	General regulatory factor 6	GRF6	FW	AGATGGACGAGGCCTGAGGATC	designed in Primer3
			RV	TGGTGGCAGAAACATCGCGTAAC	
AT5G15490	UDP-glucose dehydrogenase 3	UGD3	FW	AGGTGACAAGGCTCGTCTCAGC	designed in Primer3
			RV	TGGGCTGGAGATGAAGTGGGTG	
AT5G15970	Cold-responsive 6.6	COR6.6	FW	GAGACCAACAAGAATGCCTTCC	Rhode *et al.* 2004
			RV	TGCTCTTCTCCTCAGCTTTGC	
AT5G16050	General regulatory factor 5	GRF5	FW	CTCTTGCTGATCTGGCTCCCAC	designed in Primer3
			RV	CGAGACTACACGCACGATCAGATG	
AT5G28540	Luminal-binding protein 1	BIP1	FW	AAGCTCAAGGAGGTAGAGGCAGTG	designed in Primer3
			RV	TCGATGATTCTCCTCCTGCACCAC	
AT5G28840	GDP-mannose 3,5-epimerase	GME	FW	GGTGAGCATGAATGAGATGGCTG	designed in Primer3
			RV	CTCATATTAGGAGCCCAACCAAGC	
AT5G39320	UDP-glucose dehydrogenase 4	UGD4	FW	ACGAGCCAGGTCTTGACGATATC	designed in Primer3
			RV	TTTCCCAGCTCCAAGACCAGTC	
AT5G41210	Glutathione S-transferase THETA 1	GSTT1	FW	CAAAGAAGGATTCCAGAAGCGGCG	designed in Primer3
			RV	TCTTCAACGAACCAGTGACTGACC	
AT5G42020	Luminal-binding protein 2	BIP2	FW	TCGGGCACTGGACCTATTTAAGC	designed in Primer3
			RV	TGTTTGCTCCAAACGAGCGAGC	
AT5G49910	Chloroplast heat shock protein 70-2	CPHSC70-2	FW	ACTACTCCAACCTCCGCCTTCC	designed in Primer3
			RV	CGGTGTATCGGGAAGCGTTGTTG	
AT5G52310	Cold-regulated 78	COR78	FW	GCACCAGGCGTAACAGGTAAAC	Rhode *et al.* 2004
			RV	AAACACCTTTGTCCCTGGTGG	
AT5G54160	O-Methyltransferase 1	OMT1	FW	CACCAGACTCAAGCCTCTCAACC	designed in Primer3
			RV	CTCGGTTCGTTCTTTGCCTCCG	
AT5G65020	Annexin 2	ANNAT2	FW	TGACATGGAACGCATCAAAGAGG	designed in Primer3
			RV	TCGCCATGTCCGAGAAGAGC	
AT5G65430	General regulatory factor 8	GRF8	FW	TGCTCAGGACGTTGCAGTTG	designed in Primer3
			RV	GCCTGTTTCGCCATGCTACAAG	
AT3G18780	Actin 2	ACT2	FW	TCCCTCAGCACATTCCAGCAGAT	Skirycz *et al.* 2007
			RV	AACGATTCCTGGACCTGCCTCATC	
AT2G32170	EXPRS	AT2G32170	FW	ATCGAGCTAAGTTTGGAGGATGTAA	Czechowski *et al.* 2005
			RV	TCTCGATCACAAACCCAAAATG	
AT1G13320	PDF2	PP2AA3	FW	TAACGTGGCCAAAATGATGC	Czechowski *et al.* 2005
			RV	GTTCTCCACAACCGCTTGGT	
AT5G65080	Intron MAF5	MAF5	FW	TTTTTTGCCCCCTTCGAATC	Czechowski *et al.* 2005
			RV	ATCTTCCGCCACCACATTGTAC	
AT1G13440	5´-GAPDH	GAPC2	FW	TCTCGATCTCAATTTCGCAAAA	Czechowski *et al.* 2005
			RV	CGAAACCGTTGATTCCGATTC	
AT1G13440	3´-GAPDH	GAPC2	FW	TTGGTGACAACAGGTCAAGCA	Czechowski *et al.* 2005
			RV	AAACTTGTCGCTCAATGCAATC	
