# Supplementary figures and images for: Repair of sub-lethal freezing damage in leaves of Arabidopsis thaliana
Source: BMC Plant Biol. 2020 Jan 20;20:35. doi: 10.1186/s12870-020-2247-3 (PMC6971927; doi:10.1186/s12870-020-2247-3)

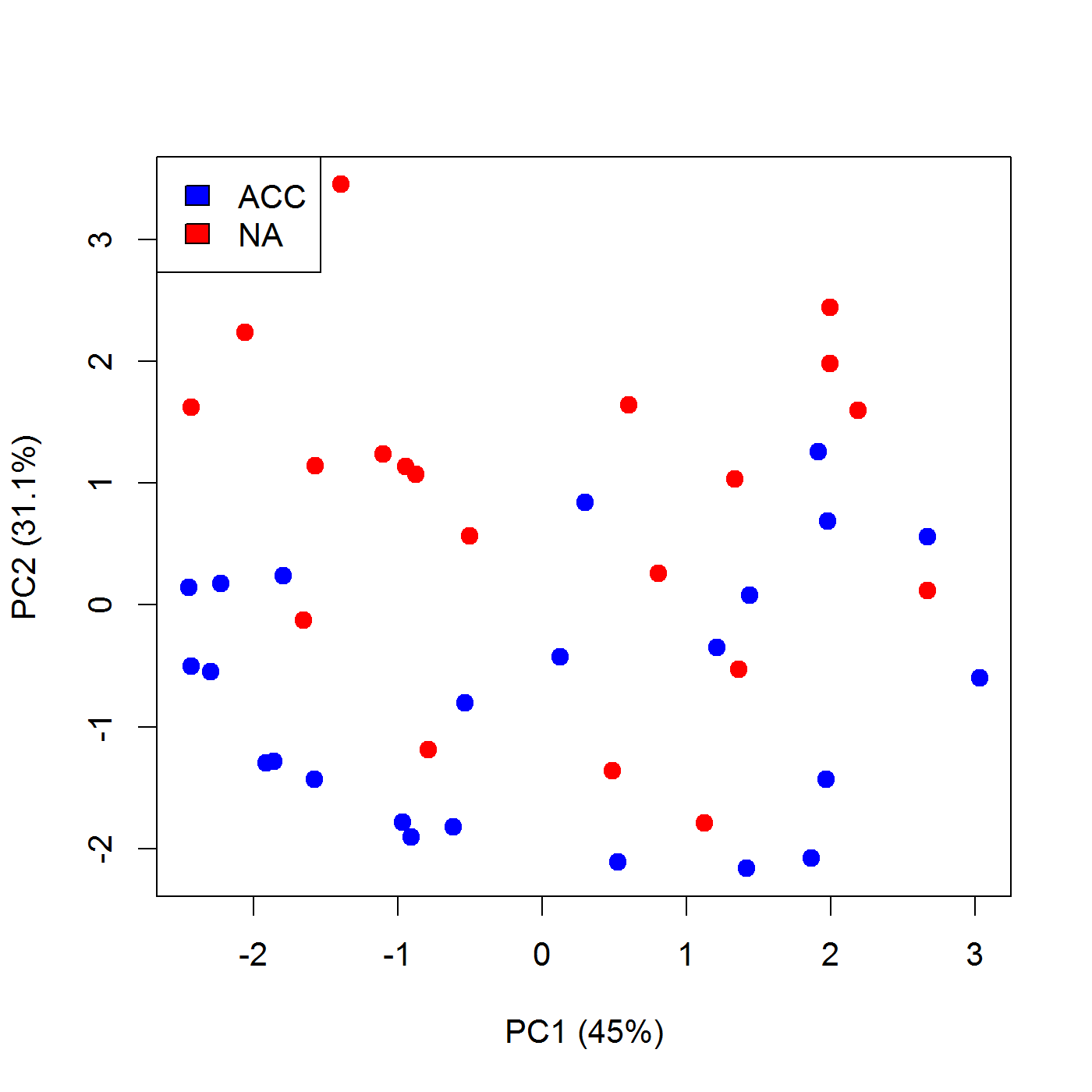

Supplement: Supplementary file 4 — Additional file 4: Figure S1. Probabilistic Principal Component Analysis (PCA) using expression data of 41 genes measured by qRT-PCR in Arabidopsis leaves frozen to different temperatures right after thawing or after recovery for 1, 3 or 6 days at 4 °C for non-acclimated (NA) plants (red dots) and cold acclimated (ACC) plants (blue dots). The mean gene expression was divided by the median gene expression across all condition and the log10 was calculated. [file 12870_2020_2247_MOESM4_ESM.tif]

## Sampling scheme non-acclimated samples

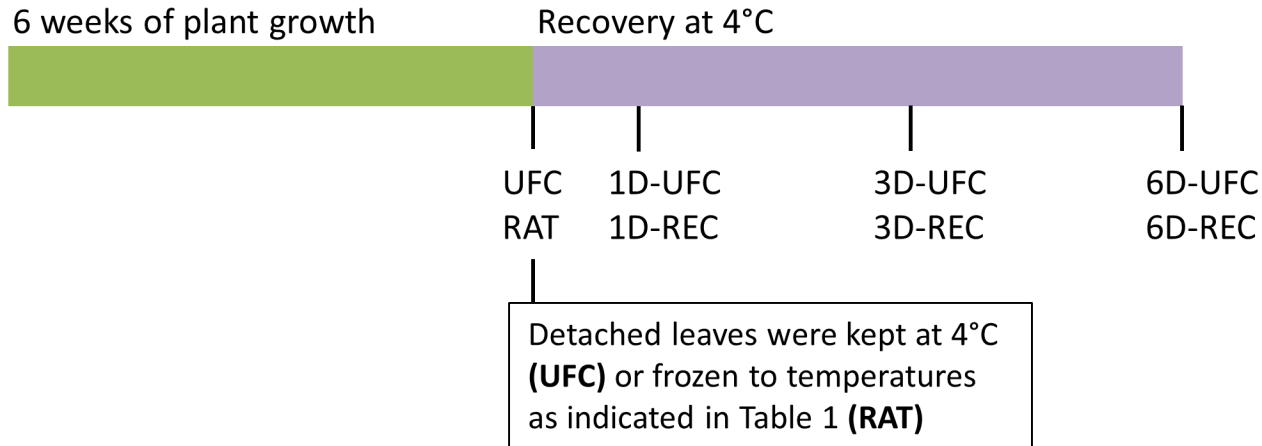

## Sampling scheme cold-acclimated samples

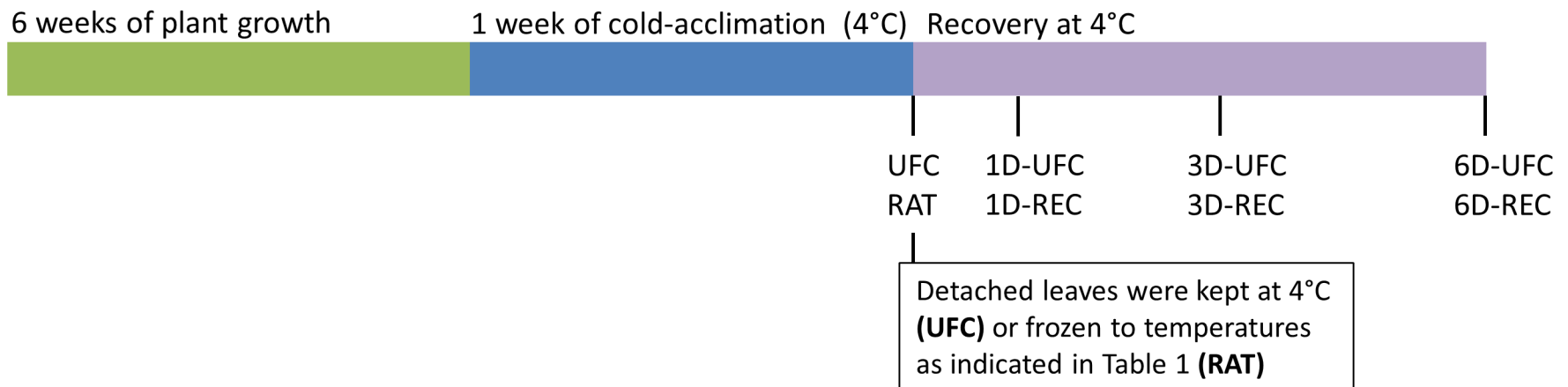

Supplement: Supplementary file 5 — Additional file 5: Figure S2. Sampling scheme for the recovery of detached leaves from non-acclimated and cold acclimated plants after freezing. RAT – right after thawing, REC – recovery, UFC – unfrozen control, D - day. [file 12870_2020_2247_MOESM5_ESM.pdf]
